# What can patients tell us in Sjögren’s syndrome?

**DOI:** 10.1515/rir-2024-0004

**Published:** 2024-03-31

**Authors:** Joe Berry, Jessica Tarn, Dennis Lendrem, John Casement, Wan-Fai Ng

**Affiliations:** Translational and Clinical Research Institute, Newcastle University, Newcastle upon Tyne, UK; National Institute for Health and Care Research (NIHR) Newcastle Biomedical Research Centre& NIHR Newcastle Clinical Research Facility, Newcastle upon Tyne Hospitals NHS Foundation Trust, Newcastle upon Tyne, UK

**Keywords:** Sjögren’s, patient-reported outcomes, electronic patient-reported outcomes, stratified medicine, heterogeneity, newcastle Sjögren’s stratification tool, endotypes, composite endpoints

## Abstract

In Sjögren’s Syndrome (SS), clinical heterogeneity and discordance between disease activity measures and patient experience are key obstacles to effective therapeutic development. Patient reported outcome measures (PROMs) are useful tools for understanding the unmet needs from the patients’ perspective and therefore they are key for the development of patient centric healthcare systems. Initial concern about the subjectivity of PROMs has given way to methodological rigour and clear guidance for the development of PROMs. To date, several studies of patient stratification using PROMs have identified similar symptom-based subgroups. There is evidence to suggest that these subgroups may represent different disease endotypes with differing responses to therapeutic interventions. Stratified medicine approaches, alongside sensitive outcome measures, have the potential to improve our understanding of SS pathobiology and therapeutic development. The inclusion of PROMs is important for the success of such approaches. In this review we discuss the opportunities of using PROMs in understanding the pathogenesis of and therapeutic development for SS.

## Introduction

There are currently no proven effective disease modifying therapies for Sjögren’s syndrome (SS). Many therapies that are effective for other immune mediated inflammatory diseases (IMIDs) have not been shown to be effective in SS. One of the main barriers to effective therapeutic developments in SS is the heterogeneity in clinical manifestations making it difficult to accurately evaluate effectiveness of therapies.^[1]^ In SS, for example, some patients present with overt inflammatory symptoms and multi-system involvement. Some report the classical dryness symptoms but other typical symptoms such as pain and fatigue may be minimal. Some patients may present with high levels of symptoms of anxiety and depression.

Patient reported outcome measures (PROMs) are standardised and validated questionnaires designed to measure the patients’ perception of their disease.^[2, 3, 4, 5]^ PROMs play an important part in the clinical management of many chronic diseases by improving patient-clinician communication and contributing to a patient-centred approach to care. There are well developed processes and guidance for the development of PROMs.

The complexity of PROMs being used varies, ranging from single item to multiple items or even in several dimensions or domains. For example, the European League Against Rheumatism (EULAR) Sjögren’s Syndrome Patient Reported Index (ESSPRI)^[6]^ score combines patients scores for Pain, Fatigue and Dryness. Similarly, some PROMS aimed at the measurement of individual symptoms may comprise of multiple items to evaluate different facets of that symptom. Examples are the mental and physical fatigue components of the Profile of Fatigue and Discomfort (PROf),^[7]^ the ocular and oral dryness components of the EULAR Sicca Scale (EULAR-SS) and the Composite Autonomic Symptom Scale (COMPASS31).^[8]^ They may be yet more complex still, capturing functional aspects of health and daily living. The EuroQol five-dimension scale questionnaire (EQ-5D)^[9]^ is widely used to combine scores on multiple dimensions to measure Quality of Life (QoL) and the Health Assessment Questionnaire (HAQ)^[10]^ is widely used to measure general health and wellbeing.

PROMs are also widely used in therapeutic development and evaluation of healthcare outcomes in medical research. ^[11, 12, 13, 14]^ PROMs can be used as stand-alone measurements or can also be combined with clinical measures of disease activity. For example, the recently developed Composite of Relevant Endpoints for Sjögren’s Syndrome (CRESS)^[15]^ and Sjögren’s Tool for Assessing Response (STAR)^[16]^ incorporate objective clinical measurements (Salivary Flow Rate, Schirmers Score, IgG titres) with clinical disease activity measures (ESSDAI)^[17]^ and PROMs (ESSPRI). In other IMIDs for example, disease activity scores – 28 joints (DAS-28) for rheumatoid arthritis (RA) include the Patient Global Visual Analogue Scale (VAS), a patient-reported score of disease activity.

In this paper, we first review how PROMs have been used for stratified medicine in SS, before analysing the relationship between PROMs and other clinic measures used in SS.

## PROMs as Tools for Stratified Medicine

It is widely recognized that a patient’s experience is inadequately captured by physician documentation, particularly regarding symptoms. PROMs highlight the clinical heterogeneity of SS and it has been established that clinical heterogeneity in SS as measured using PROMs corresponds well to patients’ health-related quality of life.^[18]^

Recently, PROMs have been used to help identify distinct pathobiological subtypes of SS. Tarn *et al*.^[19]^ were the first to report the findings of using PROMs to stratify SS patients. They used PROMs to perform the initial clustering and then test if the resulting clusters are clinically or biologically meaningful. Their study used the Pain, Fatigue and Dryness scores of the ESSPRI with Anxiety and Depression scores from the Hospital Anxiety & Depression scale (HADs)^[19]^ and reported four symptom-based subgroups of SS. In addition, they have developed an algorithm, the Newcastle Sjögren’s Stratification Tool (NSST), for researchers and clinicians to stratify any SS patient into these four subgroups (provided data on the 5 component symptom scores are available). The four NSST groups – the High Symptom Burden (HSB) and Low Symptom Burden (LSB) groups, together with a Pain-Dominated Fatigue (PDF) and Dryness-Dominated Fatigue (DDF) accounted for variation in both clinical and biological parameters. These patterns were replicated in two additional European cohorts.

In this example of symptom-based stratification the ESSPRI subdomains are crucial components when distinguishing the groupings, however in addition, measures of anxiety and depression are also essential when separating the PDF and HSB groupings. Despite being common symptoms of SS, measures of anxiety and depression are not routinely recorded in SS cohorts, and rarely are HADs data collected, which is problematic when deploying NSST in wider SS cohorts. From our own unpublished investigations, we have found surrogate measures of anxiety and depression such as associated transcriptomic variables or the EQ-5D anxiety/depression dimension are poor substitutes for HADs data in allocation of NSST membership. We argue that anxiety and depression are key symptoms of SS, separate from other related symptoms such as fatigue and should be collected routinely such that they can be utilised for symptom-based stratification.

While PROMs-based clusters look promising and appear to have biological and clinical relevance, whether these represent different pathobiological endotypes requires further evidence at the molecular level. Tarn and colleagues reported differences in the transcriptomics and cytokine expression of the HSB, LSB, PDF and DDF phenotypes in the UK Primary Sjögren’s Syndrome Registry (UKPSSR) cohort, and such differences were replicated in the Assessment of Systemic Signs and Evolution of Sjögren’s Syndrome (ASSESS) cohort.^[19]^ In addition, analysis of longitudinal data from a regional UK SS cohort suggests that NSST subgroup membership predicts long term outcomes for health-related quality of life, which has implications for cost-benefit analysis in drug development.

Analysis of comorbid conditions and medications use also revealed differences between the NSST subgroups in the UK cohort.^[20]^ Both the LSB and DDF subgroups showed lower levels of medication use and were characterised by fewer chronic comorbid conditions. In contrast, the HSB subgroup and to a lesser extent the PDF subgroup reported higher levels of polypharmacy and more chronic comorbid conditions. Peripheral vascular disease, spondylopathies and fibromyalgia were most prevalent in the HSB group whereas the PDF patients had greater prevalence of cardiovascular disease and gastrointestinal comorbidities.

In addition, reanalysis of two clinical trials stratifying by these NSST subgroups suggested differential responses to the two therapies – hydroxychloroquine and rituximab. The analysis suggests that the HSB group is more likely to show improvement in ESSPRI after hydroxychloroquine treatment compared to the other groups, whereas the DDF group is more likely to respond to rituximab therapy as measured by improvements in salivary flow rate.^[21]^ However, adequately powered stratified trials are needed to confirm these findings. These data highlight the importance of stratification in clinical trials in a heterogeneous disease such as SS.

Several other studies have reported distinct patient groupings using PROMs data. Despite using different cohorts and methodologies, including the use of a slightly different set of PROMs in the stratification approaches, the subgroups observed in these studies are broadly similar to the NSST groupings. The results of these studies are summarised in Table 1. All four studies identified SS subgroups with high disease burden, low disease burden and subgroups that were dominated by symptoms of dryness or pain. Collectively, these data substantiate the hypothesis that these symptom-based subgroups are true representation of the differences in the patients’ experience of the disease.

**Table 1:** Summary of Sjögren’s patient stratification studies using PROMs.

Study	Study population	PROMs used in stratification	Methodology	Subgroup Identified	Objective findings between strata
Tarn *et al*. ^[19]^	UK (UKPSSR, *n* = 608) France (ASSESS, *n* = 334) Norway (*n* = 62)	ESSPRI-Pain ESSPRI-Fatigue ESSPRI-Dryness HAD-A HAD-D	Hierarchical clustering Discriminant Analysis Development of NSST	4 Subgroups: LSB HSB PDF DDF	Serum IgG highest in LSB and DDF subgroups. Lowest IFN response in HSB and PDF subgroups. Increased prevalence of lymphoma in DDF subgroup. Suggestion of differential response to therapy^[21]^ Chronic comorbid conditions and polypharmacy increased in HSB and PDF subgroups including higher prevalence of fibromyalgia in HSB subgroup.^[20]^ Reduced health related-QoL particularly in HSB subgroup.^[18]^
Lee *et al*. ^[22]^	KISS (*n* = 321)	ESSPRI-Pain ESSPRI-Fatigue ESSPRI-Dryness	Latent class Analysis	3 Subgroups: LSB HSB DD	Differences in SSDDI and unstimulated salivary flow between the groups. EQ-5D anxiety and depression subscale High stability of subgroup membership over 5 years follow up.
McCoy *et al*.^[23]^	SICCA (*n* = 1454) US Sjögren’s Foundation Survey (*n* = 2920)	ESSPRI-Pain ESSPRI-Fatigue ESSPRI-Dryness	Hierarchical Clustering	4 Subgroups: LSB HSB DHP DLP	IgG, WCC highest in LSB and DLP subgroup. Milder organ manifestations in HSB subgroup Higher medication costs in HSB subgroup Higher prevalence of fibromyalgia in HSB subgroup
Gairy *et al*.^[24]^	US, Italy, Spain, Germany, France (*n* = 316)	ESSPRI-Pain ESSPRI-Fatigue Organ manifestations based on clinician assessment of ESSDAI subdomains	Latent Class analysis	5 Subgroups: Low burden Low burden (articular) Moderate burden (articular) Moderate burden (multi-organ) High burden (multiorgan)	High burden (multi-organ) subgroup associated with highest level of treatment burden.

PROMs, Patient Reported Outcome Measures; EULAR, European League Against Rheumatism; ESSPRI, EULAR Sjögren’s Syndrome Patient Reported Index; UKPSSR, UK Primary Sjögren’s Syndrome Registry; ASSESS, Assessment of Systemic Signs and Evolution of Sjögren’s Syndrome; KISS, Korean Initiative Sjögren’s Syndrome; SICCA, Sjögren’s International Collaborative Clinical Alliance; ESSDAI, EULAR Sjögren’s Syndrome Disease Activity Index; HAD-A, Hospital Anxiety Depression Scale-Anxiety; HAD-D: Hospital Anxiety Depression Scale-Depression; NSST, Newcastle Sjögren’s Stratification Tool; LSB, Low Symptom Burden; HSB, High Symptom Burden; PDF, Pain Dominated with Fatigue, DDF, Dryness Dominated with Fatigue; DD, Dryness Dominant; DHP, Dry High Pain; DLP, Dry Low Pain; SSDDI, Sjögren’s Syndrome Disease Damage Index; IgG, Immunoglobulin G; IFN, Interferon; EQ-5D, EuroQual 5 Dimensions; WCC, White Cell Count.

## Exploring the Relationships between Objective and Subjective Clinical Measures in SS

To explore the relationship between objectively measured parameters (clinical scores, laboratory data) and PROMs, we took advantage of the data available from a large cohort of well-characterised SS patients from the UKPSSR.^[25]^ To assess concordance between objective and subjective clinical measures in SS we used the ARACNE (Algorithm for the Reconstruction of Accurate Cellular Networks) algorithm, a network reconstruction approach.^[26]^ ARACNE calculates degrees of mutual information (MI) between variables of a multidimensional dataset and can be used to reflect shared information and correlation between variables within a network.

**Figure 1:**
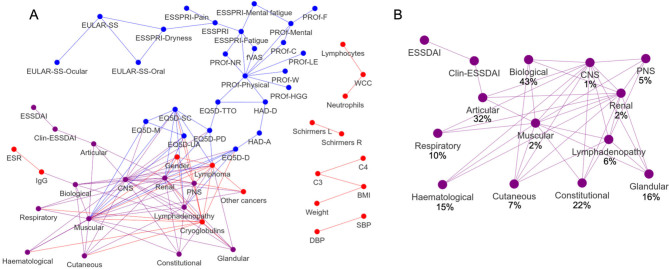
A) ARACNE Clinical network reconstruction for a cross-sectional dataset of 624 patient reported outcome measures and objective clinical and laboratory observations in SS. Edges between nodes represent shared information between the nodes. B) Sub-network of the ESSDAI subdomains showing the connections between them. Percentages in brackets represent the proportion of non-zero scores for each domain. Abbreviations: fVAS, Fatigue Visual Analogue Scale; BMI, Body Mass Index; Clin-ESSDAI, Clinical ESSDAI; CK, Creatin Kinase; Other_cancers, Current / Historical Cancer (not lymphoma); Lymphoma, Current / Historical Lymphoma; EQ-5D-D, EQ-5D- Depression; EQ-5D-M, EQ-5D- Mobility; EQ-5D-PD, EQ-5D- Pain/Discomfort; EQ-5D-SC, EQ-5D- Self Care; EQ-5D-UA, EQ-5D- Usual Activities; ESR, Erythrocyte Sendimentation Rate; ESSPRI, EULAR Sjögren’s Syndrome Patient Reported Index; ESSDAI, European League Against Rheumatism (EULAR) Sjögren’s Syndrome Disease Activity Index; Articular, ESSDAI- Articular Domain; Biological, ESSDAI- Biological Domain; CNS, ESSDAI- Central Nervous System Domain; Constitutional, ESSDAI- Constitutional Domain; Cutaneous, ESSDAI- Cutaneous Domain; Glandular, ESSDAI- Glandular Domain; Haematological, ESSDAI- Haematological Domain; Lymphadenopathy, ESSDAI- Lymphadenopathy Domain; Muscular, ESSDAI- Muscular Domain; PNS, ESSDAI- Peripheral Nervous System Domain; Renal, ESSDAI- Renal Domain; Respiratory, ESSDAI- Respiratory Domain; EULAR-SS, EULAR Sicca Scale; EULAR-SS-Ocular, EULAR Sicca Scale- Ocular; EULAR-SS-Oral, EULAR Sicca Scale- Oral; EQ-5D-TTO, EuroQual 5 Dimensions (EQ-5D) - Time Trade Off; HAD-A, Hospital Anxiety Depression Scale- Anxiety; HAD-D, Hospital Anxiety Depression Scale-Depression; OSF, Oral Salivary Flow; Plt, Platelet Count; PROf-Mental, Profile of Fatigue (PROf) - Mental Fatigue; PROf-Physical, PROf- Physical Fatigue; PROf-C, PROf- Concentration; PROf-F, PROf- Forget; PROf-HGG, PROf- Hard to Get Going; PROf-LE, PROf- Lacking Energy; PROf-NR, PROf- Need Rest; PROf-W, PROf- Weak; Schirmers_L, Schirmer’s Left Eye; Schirmers_R, Schirmer’s Right Eye; SBP, Systolic Blood Pressure; DBP, Diastolic Blood Pressure; WCC, White Cell Count.

Figure 1 is a network representation of the relationships between clinical variables in a cohort of 624 primary SS patients from the UKPSSR. The network illustrates the relationships between the key clinical measures, laboratory data and PROMs and may help us to understand the potential interactions between them.

Nodes within the clinical network represent each of the variables and the edges between them represent the relationship between those variables. Edges are filtered to those that have significant (*P* < 1^–10^) mutual information as determined by the ARACNE algorithm. Therefore, where nodes are connected by an edge represent significant level of association whereas as nodes that are not connected by an edge suggest no significant level of association. See supplementary methods (SM1) for the ARACNE algorithm.

The resultant ARACNE network highlights key areas of concordance and discordance between PROMs, clinician-based and laboratory-based measurements. As anticipated, multi-domain PROMs and ESSDAI subdomains are strongly associated with their component scores. This observation is anticipated and provides some assurance to this approach. We also observe that different PROMs measuring the same symptoms were also correlated. For example, questionnaires related to dryness, mental fatigue (‘mental total’ from PROFAD and ‘mental fatigue’ from ESSPRI), and physical fatigue (Abnormal Fatigue (VAS), fatigue (ESSPRI), Physical fatigue (PROFAD) are well connected.

The strong relationships between ESSDAI components that are clinically unrelated may be due to the sparse nature of the data; for example, the CNS, muscular and renal domains (Figure 1B). This is because for many of the ESSDAI components, a score of 0 is proportionally the most common score, therefore the mutual information between these components is high. Nonetheless, some associations between ESSDAI domains may be of clinical relevance. For instance, the glandular, lymphadenopathy and renal domains are associated with each other. Glandular swelling, lymphadenopathy and renal manifestations are known risk factors for lymphoma development in SS.^[27]^ Of note, BMI correlated with C3 and C4 levels but has a weak relationship with other clinical measures, this observation is consistent with the literature that high BMI is associated with elevated C3 levels.^[28]^

In contrast, there is a clear separation between PROMs, clinician-based and laboratory-based measurements (e. g. Lymphocytes, Neutrophils, WCC), each forming their own sub-networks or fragmented networks. Furthermore, some-what surprisingly, most of the laboratory measurements do not have strong relationship with clinician-based disease activity score or PROMs. An interesting example of this discordance is the biological domain having a weak association with total ESSDAI score, with no direct edges between them. Another example is the weak association between objective measures of ocular dryness, such as Schirmer’s score, and the patient experience of ocular dryness; although this is not entirely unexpected given previous reports of discordance between objective tear production and experience of ocular dryness.^[29]^ In contrast, the articular domain is the only domain directly associated with clin-ESSDAI and indirectly with ESSDAI which likely is representative of the higher frequency with which patients have an articular domain score > 0 (32%) and the higher weighting of this domain for ESSDAI score calculation.

In SS therefore, it is evident that objective clinical measures of disease activity such as clinical laboratory parameters, disease activity indices such as ESSDAI or specific measures of systemic involvement correlate poorly with PROMs such as ESSPRI.^[30,31]^ A plausible explanation is that patients’ symptoms and the objective systemic manifestations may represent two different components of the disease that are driven by different underpinning mechanisms, and therefore necessitate separate evaluation. Even for cardinal symptoms such as oral and ocular dryness while there is evidence for an association between objective measures and patient-reported dryness scores,^[32,33]^ there is also considerable discrepancy between objective and subjective measures among many SS patients.^[34]^ More recently researchers have developed composite disease assessment tools incorporating PROMs and objective clinical measures to create more clinically meaningful endpoints for SS trials, namely the CRESS^[15]^ and STAR.^[16]^ These composite endpoints are being evaluated for use in future clinical trial development alongside stratified recruitment approaches.

Despite the lack of association between PROMS, clinician-based and laboratory-based measurements, both the ESSDAI and ESSPRI and their components are related to EQ-5D utility values and thus are both important contributors to QoL. In addition, symptoms of depression and pain/discomfort are associated with the CNS, Renal and Lymphadenopathy sub-domains of the ESSDAI score. In our assessment the HR-QoL metrics provide the primary links between the ESSPRI and ESSDAI subdomain scores.

The dissociation between PROMs and objective clinical measures raises important issues. Firstly, it complicates clinical management of SS including any treat-to-target strategy. Secondly, its highlights importance of PROMs in supporting clinician assessment and laboratory measurements to better capture all aspects of SS and give a more individualised and meaningful assessment. Finally, it supports the dual-target strategy of systemically pursuing two targets in the design of clinical trials: one focused on the disease activity (often with known candidate biological targets) and another focused on the symptoms and impact (the impact target).^[35,36]^

## Discussion: The future benefits of patient reported outcomes

At present, there are no proven treatments that slow disease progression or treat the systemic manifestations of SS.^[37]^ The failure of many previous clinical trials in SS may be explained by clinical heterogeneity, knowledge gaps in understanding of the pathophysiology, insensitive outcome measures, and a robust placebo response.^[38]^ The patient experience varies widely and may reflect disease heterogeneity rather than disease severity or disease activity.

Stratified medicine techniques, alongside careful selection of relevant and sensitive outcome measures, are expected to provide key insights into pathobiology and improve clinical trial design in SS. The inclusion of PROMs is vital for the success of such approaches. PROMs-based stratification also allow for patient stratification at the point of care and by accompanying more conventional clinical trial end-points allow researchers to better capture all the aspects of SS. The recent developments in composite endpoints, such as CRESS and STAR, may enable a more specific and meaningful assessment of intervention or therapeutics in future trials.

The symptomatology of SS is likely underpinned by a network of dysregulated molecular pathways. PROM-based stratification may also highlight potentially important molecular pathways contributing to the pathogenesis of SS and help us to better understand the biological basis of clinical heterogeneity in SS, as illustrated by the NSST subgroups.

With the advent of composite endpoints and PROM-based stratification tools, methodological rigour when developing and utilising PROMs have become even more important. A fuller understanding of measurement variability and factors affecting PROMs is essential to interpreting longitudinal changes or differences between studies. For example, longitudinal studies suggest that ESSPRI scores for patients are relatively stable over time, could suggest that ESSPRI is insensitive to change.^[26]^ However, it remains possible that with appropriate effective treatment, ESSPRI improvement can be observed. Recent work using salivary gland ultrasound (SGUS) provided more insight into ESSPRI, showing that patients who were SGUS-positive scored significantly lower on the ESSPRI for fatigue and pain, and more often found their disease state acceptable compared with SGUS-negative patients.^[28]^ Given the significant clinical heterogeneity and growing use of PROMs in SS, time invested in understanding sources of variability in proposed outcome measures is not wasted time.

The adoption of artificial intelligence (AI) technology in healthcare research is transforming the field, and researchers are beginning to understand the importance of including PROMs in AI models alongside objective clinical and experimental measurements.^[39,40]^ Looking forward, frequent electronic monitoring of PROMs (ePROMs) could be a unique solution to improving patient care in SS, and this approach has already been adopted with success in the field of oncology.^[41]^ The use of ePROs in patients with cancer has been shown to improve clinical outcomes, including symptom control, better quality of life, decreased emergency department visits/ hospitalizations, and longer survival. Not only this, but patients have also stated they feel greater control of their care and more connected to their care team with ePROMs without negative aspects of social media.^[42]^

ePROMs address problems with inter-individual variability of symptoms and when correctly designed may capture a broad range of effects of immune dysfunction. Such information could potentially help clinicians to detect disease flares early and avert preventable downstream complications. In addition, frequent collection of symptom-based data could lead to a greater understanding of symptom variability.

## Conclusions

PROMs are important tools for the development and provision of patient-centric health services. In SS, the significant heterogeneity in clinical manifestations and a discordance between ‘objective’ clinical measures and PROMs has led to the use of composite outcome measures combining both conventional endpoints and PROMs. Recent studies have shown that PROMS can be useful for stratified medicine and the findings between these studies corroborate each other. These studies also suggest that heterogeneity in PROMs may reflect distinct pathobiological endotypes. Better understanding of the mechanisms underpinning the heterogeneity in clinical manifestations and PROMS may be critical to the development of effective therapeutics and precision medicine for SS.
